# Vedolizumab-Induced Interstitial Lung Disease: A Case of Delayed-Onset Pulmonary Toxicity

**DOI:** 10.7759/cureus.96601

**Published:** 2025-11-11

**Authors:** Tania Lasrado, Qais Akasheh, Shabnam Enam, Matthew Burton

**Affiliations:** 1 Respiratory Medicine, East Suffolk and North Essex NHS Foundation Trust, Ipswich, GBR

**Keywords:** drug-induced lung injury, hypersensitivity pneumonitis, inflammatory bowel disease, interstitial lung disease, vedolizumab

## Abstract

Vedolizumab, a monoclonal antibody targeting α4β7 integrin, is widely used for the treatment of moderate to severe inflammatory bowel disease (IBD) and is generally well tolerated. Pulmonary toxicity is exceedingly rare, with only a small number of interstitial lung disease (ILD) cases reported.

We describe a 46-year-old woman with ulcerative colitis (UC), managed with vedolizumab for two years, who developed progressive dyspnea and cough. Despite corticosteroid therapy, her symptoms worsened. Imaging demonstrated diffuse centrilobular nodules consistent with hypersensitivity pneumonitis. Infectious, autoimmune, and other secondary causes were excluded. Following another vedolizumab infusion, her symptoms deteriorated further. Vedolizumab was permanently discontinued, leading to marked clinical, functional, and radiological improvement within months.

Drug-induced ILD is a challenging diagnosis due to its nonspecific clinical and radiological features. In this case, the temporal association, exclusion of alternative causes, relapse on re-challenge, and improvement after drug withdrawal strongly supported vedolizumab-induced ILD. A review of published literature confirms this as a rare but potentially serious adverse effect.

Vedolizumab-induced ILD, though uncommon, should be considered in patients presenting with new-onset respiratory symptoms during biologic therapy. Early recognition and discontinuation of the offending drug are critical to prevent long-term pulmonary complications.

## Introduction

Vedolizumab (Entyvio) is a biologic that targets gut-specific integrins and is commonly used to treat moderate to severe ulcerative colitis (UC) and Crohn’s disease. It is generally well tolerated, with known side effects including respiratory symptoms such as cough, sinus infections, and bronchitis, as well as infusion reactions [1]. However, interstitial lung disease (ILD) related to vedolizumab is extremely rare, only about 10 cases have been documented.

In recent years, the number of medications linked to drug-induced interstitial lung disease (DIILD) has steadily increased, particularly with the introduction of monoclonal antibodies and biologic therapies for cancer and autoimmune conditions. These agents include chemotherapeutics, immune checkpoint inhibitors, antibiotics, antiarrhythmics, and both traditional and biologic disease-modifying antirheumatic drugs. The severity of DIILD can range from mild symptoms to life-threatening respiratory failure [2,3].

Here, we describe a case of vedolizumab-associated ILD that fully resolved following discontinuation of the drug.

## Case presentation

We present the case of a 46-year-old woman with a long history of ulcerative colitis, diagnosed at age 8. She also had asthma and Gilbert’s syndrome. Her UC was well managed with azathioprine and mesalazine for many years; however, in 2018, she began experiencing severe flares that did not respond to steroids. After an unsuccessful trial of infliximab, vedolizumab was initiated. The treatment worked initially, but she paused therapy in September 2019 during her third trimester of pregnancy.

**Figure 1:**
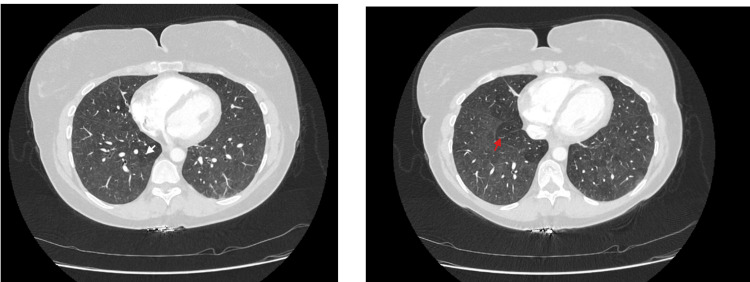
CT thorax with contrast while on vedolizumab therapy. White and red arrows indicate areas of air trapping.

When her symptoms flared again in 2021, vedolizumab was restarted. Two years later, in September 2023, she developed new respiratory symptoms, shortness of breath on exertion and intermittent cough. Despite several courses of corticosteroids, her symptoms did not improve. A CT pulmonary angiogram in July 2024 revealed widespread low-density centrilobular nodules in both lungs, a pattern consistent with acute hypersensitivity pneumonitis or a drug reaction (Figure 1) [3]. There was no evidence of fibrosis, and serologic workup was unrevealing (Table 1).

**Table 1:** Blood test results over the clinical course.

Parameter	Reference Values	September 6, 2023	October 16, 2023	March 11, 2024	April 23, 2024	July 1, 2024	January 29, 2025
CRP (mg/L)	<5	9	19	7	5	10	<1
Haemoglobin (g/L)	115-165 (women)	132	138	136	133	145	140
White cell count (×10⁹/L)	3.6-11.0	6.5	7.7	7.2	6.3	11.5	6.7
Neutrophils (×10⁹/L)	1.8-7.5	4.7	6.2	5.9	4.6	4.6	4.6
Lymphocytes (×10⁹/L)	1.0-4.0	1.1	0.7	0.8	1.1	1	1.5
Eosinophils (×10⁹/L)	0.1-0.4	0.2	0.3	0.2	0.2	0.2	0.1
Monocytes (×10⁹/L)	0.2-0.8	0.4	0.4	0.3	0.5	0.4	0.6
ESR (mm/h)	<20	8	12	12	-	17	-

A review of the literature and the Pneumotox database identified both mesalazine and vedolizumab as potential culprits for drug-induced pneumonitis, although mesalazine reactions typically occur after long-term exposure [2,3]. The patient was started on 40 mg of oral prednisolone, which was tapered to 30 mg without improvement. After another vedolizumab infusion, her breathing worsened within a week, possibly exacerbated by the reduced steroid dose.

**Figure 2:**
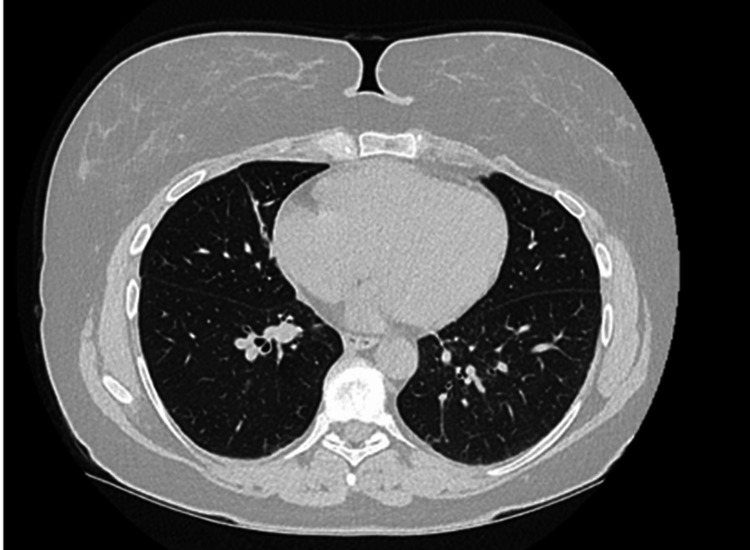
CT thorax with contrast after discontinuation of vedolizumab showing resolution of interstitial changes.

Vedolizumab was permanently discontinued in September 2024. Within a month, her breathing improved significantly, and lung function tests confirmed progress. By December 2024, she remained improved even on lower steroid doses. A high-resolution CT scan in March 2025 showed no evidence of ILD or fibrosis (Figure 2). She continues azathioprine and mesalazine for UC and remains clinically stable.

## Discussion

DIILD is a challenging and often elusive diagnosis [2,3]. It occurs when a medication triggers inflammation, and in some cases, fibrosis, of the lung interstitium. The clinical, radiological, and pathological features of DIILD are often non-specific, making it difficult to distinguish from other ILDs [2,3]. Because symptoms and imaging findings vary widely between drugs and even among patients on the same drug, DIILD remains a diagnosis of exclusion [2,3]. Diagnosis is based on compatible ILD findings, a temporal link to drug exposure, and the absence of alternative causes such as infection, pulmonary oedema, radiation injury, or disease progression. Improvement after drug withdrawal, sometimes with corticosteroids, and deterioration on re-exposure support the diagnosis [2,3].

Vedolizumab is a monoclonal antibody targeting the α4β7 integrin to prevent leucocyte infiltration into the gastrointestinal submucosa [1]. Long-term trials have demonstrated a favourable safety profile [4,5]. It is not associated with an increased risk of serious or opportunistic infections, and malignancy rates are similar to background IBD rates. Infusion reactions, enteric infections, and autoimmune events are infrequent [4].

In recent years, non-infectious pulmonary injury linked to vedolizumab has been reported. Symptom onset varies from as early as after two doses [6] to several years after initiation [7,8]. In our patient, respiratory symptoms began after two years of treatment.

Recent reports have described various non-infectious pulmonary complications associated with vedolizumab, including hypersensitivity pneumonitis, organizing pneumonia, and necrobiotic pulmonary nodules. These cases have occurred in both adults and children with IBD. Some patients developed atypical extra-intestinal manifestations involving the lungs, while others presented with drug-related pneumonitis. Although rare, vedolizumab-induced ILD is an important adverse event that requires early recognition, discontinuation of the drug, and corticosteroid therapy for recovery [9-11].

We diagnosed vedolizumab-induced ILD based on the presence of new respiratory symptoms accompanied by imaging findings suggestive of hypersensitivity pneumonitis or a drug-related reaction, after excluding other potential causes. The diagnosis was further supported by symptomatic, functional, and radiological improvement following discontinuation of vedolizumab and initiation of corticosteroid therapy, as well as relapse upon re-challenge with the drug.

Our findings support previous reports that vedolizumab-induced ILD can be severe and potentially fatal, but early recognition, drug cessation, and corticosteroid use (when indicated) can lead to recovery.

## Conclusions

Vedolizumab-induced ILD is an exceedingly rare but important differential diagnosis in patients presenting with new-onset respiratory symptoms while receiving biologic therapy for IBD. This case underscores the need for heightened clinical suspicion, particularly when symptoms are unresponsive to corticosteroids and imaging suggests a hypersensitivity or drug-related pattern. Prompt discontinuation of the suspected agent, with or without corticosteroid therapy, can lead to significant clinical and functional improvement. As the use of vedolizumab continues to expand, clinicians should remain vigilant about this potential adverse effect to enable early recognition and prevent long-term pulmonary complications.
